# Clinical characteristics and risk factors of acute cutaneous graft-vs.-host disease following allogeneic hematopoietic stem cell transplantation in pediatric acute myeloid leukemia: a single-center retrospective study

**DOI:** 10.3389/fped.2026.1820755

**Published:** 2026-07-23

**Authors:** Shan He, Mincui Zheng, Wenyong Kuang, Na Song, Shaoyang Deng, Benshan Zhang

**Affiliations:** Department of Hematology/Oncology, The Affiliated Children’s Hospital of Xiangya School of Medicine, Central South University (Hunan Children’s Hospital), Changsha, China

**Keywords:** acute myeloid leukemia, graft-vs.-host disease, hematopoietic stem cell transplantation, pediatric, risk factors

## Abstract

**Objective:**

To analyze clinical characteristics and risk factors of acute cutaneous graft-vs.-host disease (GVHD) in pediatric acute myeloid leukemia (AML) patients undergoing allogeneic hematopoietic stem cell transplantation (allo-HSCT), providing evidence for clinical risk stratification and individualized prophylaxis.

**Methods:**

A retrospective cohort study included pediatric AML patients who underwent their first allo-HSCT at Hunan Children's Hospital between December 2019 and September 2025. Demographic, disease-related, donor-recipient matching, transplant procedure, and supportive care data were collected. The primary outcome was acute cutaneous GVHD within 100 days post-transplant. Univariate analysis identified potential risk factors, and multivariate logistic regression confirmed independent predictors, with sensitivity analysis verifying robustness.

**Results:**

Acute cutaneous GVHD occurred in 27 pediatric patients (38.6%), including grade I (14.8%, 4/27), II (59.3%, 16/27), and III (25.9%, 7/27) cases. Multivariate analysis showed HLA mismatch at ≥2/10 alleles (OR = 4.26, 95%CI:1.68–10.82, *P* = 0.002), refractory/relapsed disease pre-transplant (OR = 6.83, 95%CI:1.57–29.65, *P* = 0.011), and myeloablative conditioning (MAC) (OR = 3.15, 95%CI:1.08–9.19, *P* = 0.036) were independent risk factors. Sensitivity analysis confirmed result robustness, and subgroup analysis showed stronger associations with grade II-III aGVHD.

**Conclusion:**

Poor HLA matching, refractory/relapsed disease pre-transplant, and MAC increase the risk of acute cutaneous GVHD in pediatric AML patients after allo-HSCT. The risk factors identified in this study are helpful for identifying high-risk pediatric AML patients before transplantation, providing a basis for implementing individualized GVHD prevention strategies.

## Introduction

Acute myeloid leukemia (AML) is a hematologic malignancy characterized by the rapid proliferation of abnormally differentiated myeloid precursors, leading to bone marrow failure (1). It is reported that the incidence of AML has been continuously rising worldwide, from 79,372 cases in 1990 to 144,645 cases in 2021 (2). The cornerstone of AML treatment has always been intensive induction chemotherapy, but a considerable proportion of patients, especially those with high-risk cytogenetics or recurrence, have a poor prognosis with chemotherapy alone (3). Allogeneic hematopoietic stem cell transplantation (allo-HSCT) has emerged as a potentially curative therapeutic modality for these individuals (4). However, the clinical benefit of allo-HSCT is largely limited by transplant-related complications, among which graft-vs.-host disease (GVHD) is the most prevalent and severe (5).

GVHD occurs when donor-derived immunocompetent cells recognize the recipient's tissues as foreign and initiate an immune response, leading to tissue damage (6). According to the onset time and clinical manifestations, GVHD is usually divided into two forms: acute GVHD (aGVHD, before 100 days after transplantation) and chronic GVHD (cGVHD, after 100 days after transplantation) (7). aGVHD mainly occurs in the skin, liver and gastrointestinal tract, among which skin involvement is often the earliest sign of the disease. Cutaneous GVHD typically presents as maculopapular rash, erythema, and pruritus in the early stage, and may progress to bullae, skin exfoliation, or even extensive cutaneous necrosis in severe cases (8). The onset of skin involvement not only necessitates immediate clinical intervention, often with high-dose systemic steroids, but also serves as a critical indicator of the overall alloreactive immune response. Its severity, graded from I to IV, is a well-established predictor of patient survival and quality of life (9). Therefore, identifying patients at high risk for developing significant (Grade II–IV) acute cutaneous GVHD before its clinical manifestation is of paramount importance. Such risk stratification could enable more personalized prophylactic strategies, enhance preemptive monitoring, and ultimately improve transplant outcomes.

Previous studies have explored risk factors for cutaneous GVHD, including donor-recipient human leukocyte antigen (HLA) matching status, donor source, conditioning intensity, and GVHD prophylaxis regimens (10–13). Nevertheless, existing research has several limitations: most studies included mixed hematological malignancies rather than focusing on AML specifically (10, 11). Additionally, previous studies on risk factors for acute cutaneous GVHD have either focused on adult populations or combined pediatric and adult patients in their analyses, potentially obscuring age-specific risk profiles and limiting the applicability of their findings to the pediatric setting (12, 13). Furthermore, the unique biological characteristics of AML, such as cytogenetic risk stratification and disease status at transplantation, may modulate the risk of cutaneous GVHD, but these factors have not been systematically evaluated in previous analyses (11, 13).

To address these gaps, we conducted this retrospective study. We hypothesized that, in pediatric AML patients undergoing allo-HSCT, the risk of developing acute cutaneous GVHD is significantly influenced by a combination of donor-recipient immunogenetic compatibility, the underlying disease biology and inflammatory state at the time of transplant, and the intensity of the transplant preparative regimen. The primary aim of this study was to test this overarching hypothesis by systematically evaluating the associations between these key domains (operationalized as specific clinical variables) and the incidence and severity of acute cutaneous GVHD. Rather than seeking to identify novel risk factors, our study provides rigorous, population-specific validation of established GVHD risk determinants within a historically understudied, homogeneous pediatric AML cohort—evidence that is essential to refine risk stratification frameworks for this distinct patient population.

## Materials and methods

### Study design and setting

This was a single-center, retrospective observational cohort study conducted at Hunan Children's Hospital. The study protocol was approved by the Ethics Committee of Hunan Children's Hospital (Approval number: KYSQ2022-155), and the requirement for informed consent was waived due to the retrospective nature of the study, in accordance with the Declaration of Helsinki. Data were extracted from the hospital's electronic medical record system and the transplantation registry database, with strict adherence to patient privacy protection regulations.

### Study population

The study cohort consisted of consecutive pediatric patients diagnosed with AML who underwent their first allo-HSCT at our institution between December 2019 and September 2025. Inclusion Criteria: (1) age < 18 years; (2) Confirmed diagnosis of AML in accordance with the Fifth Edition of the World Health Organization (WHO) Classification of Tumors of the Hematopoietic and Lymphoid Tissues (14); (3) Complete medical records available, including demographic information, AML cytogenetic and molecular genetic data, transplant-related details, post-transplant follow-up records, and outcome information; (4) Follow-up duration ≥100 days after allo-HSCT (consistent with the time window for defining acute GVHD); (5) No history of other malignant tumors or severe autoimmune diseases before transplantation. Exclusion Criteria: (1) Secondary AML, such as transformed from myelodysplastic syndrome or after chemotherapy/radiotherapy for other diseases; (2) Received haploidentical allo-HSCT with incomplete donor-recipient HLA matching data; (3) Pre-existing skin diseases that could interfere with the diagnosis of acute cutaneous GVHD; (4) Received immune-modulating therapy within 3 months before allo-HSCT; (5) Multiple allo-HSCT procedures.

### Sample size

Given the retrospective, exploratory nature of this study, a formal prospective sample size calculation for hypothesis testing was not the primary driver of patient enrollment. The study cohort included all consecutive pediatric AML patients who underwent their first allo-HSCT at our center during the predefined study period, which yielded 70 eligible patients. However, to assess the potential statistical robustness of our cohort for detecting clinically meaningful associations in the planned multivariate analyses, we performed a *post-hoc* sample size estimation using G *Power 3.1 software. This estimation was based on the primary outcome (incidence of acute cutaneous GVHD). Parameters were derived from previous literature and preliminary institutional data (15, 16), assuming an incidence of 55% in a hypothetical higher-risk group and 25% in a lower-risk group, with an alpha of 0.05, power of 0.80, and an allocation ratio of 1:1.5. This calculation suggested a minimum sample size of approximately 62 patients to detect such a difference. Our final cohort of 70 patients exceeds this estimate, providing reassurance that the study, while exploratory, has adequate sample size to identify potential risk factors of moderate to large effect size with reasonable statistical precision, and to support the logistic regression analyses performed. This approach aligns with recommendations for reporting sample size considerations in observational studies.

A *post-hoc* power calculation was conducted via G*Power 3.1 software based on previously reported incidence rates of acute cutaneous GVHD (55% in high-risk groups vs. 25% in low-risk groups). A minimal sample size of 62 patients was estimated to detect such an intergroup difference, and 70 eligible patients were ultimately enrolled to meet this threshold. Nevertheless, several constraints linked to the sample composition were recognized. Only 27 incident cases of acute cutaneous GVHD were captured in the total cohort, and extremely small sample sizes were observed in specific subgroups, including patients with refractory/relapsed disease (*n* = 5) and those with grade III cutaneous aGVHD (*n* = 7). The stability of the multivariate logistic regression model was potentially compromised by limited event numbers, and statistical precision was restricted for stratified and interaction analyses. Wide 95% confidence intervals of certain odds ratios, such as the 95% CI of 1.57–29.65 for refractory/relapsed disease, were attributed to insufficient subgroup sample sizes. Fine-grained subgroup stratification and interaction term testing were therefore not performed in the present analysis.

### Data collection

Data were systematically extracted from the hospital's electronic medical record system and transplantation registry database by two independent researchers (blinded to the study hypothesis). Discrepancies were resolved through discussion with a third senior researcher. The selection of the variables for analysis was based on a comprehensive review of the existing literature on GVHD risk factors, which implicates factors across donor-recipient matching, disease biology, transplant procedure, and recipient characteristics (10–13). Several potential confounding variables reported in previous GVHD investigations, such as detailed counts of graft T-cell subsets, precise trough concentrations of immunosuppressive agents, kinetic data of HHV-6 or CMV reactivation, and KIR ligand mismatches between donors and recipients, were not routinely archived in the institutional transplant registry throughout the study period. Residual unmeasured confounding may therefore be introduced into retrospective analyses. All clinical data were extracted uniformly by two independent researchers according to standardized variable definitions, and inconsistency during retrospective data abstraction was minimized accordingly. The collected variables included:

1. Demographic and clinical characteristics​: age at transplantation, gender, body mass index (BMI), AML subtype ]French-American-British [FAB] classification 17), disease status at transplantation (first complete remission [CR1], second complete remission [CR2], refractory/relapsed), history of prior chemotherapy or radiotherapy, and comorbidities (assessed by the Charlson Comorbidity Index [CCI] (18)). CR1 was defined as the attainment of complete remission (typically characterized by <5% blasts in the bone marrow, recovery of peripheral blood counts, and no extramedullary disease) following the first course of induction chemotherapy. CR2​ referred to the achievement of a new complete remission after the patient has experienced a relapse from CR1 (19). Refractory disease​ described the failure to achieve CR1 after initial induction therapy. Relapsed disease indicated the return of the disease after a period of remission.
2. Donor and graft-related factors​: Donor type [HLA-identical sibling donor [ISD], HLA-matched unrelated donor [MUD], haploidentical donor], donor-recipient HLA matching status (HLA-A, -B, -C, -DRB1, -DQB1 alleles; matched defined as ≥8/10 alleles), donor gender, donor age, donor-recipient ABO blood group compatibility (compatible, minor incompatible, major incompatible, bidirectional incompatible), stem cell source [peripheral blood stem cells [PBSC], bone marrow [BM], cord blood], infused total nucleated cell (TNC) dose (×10^8^/kg recipient weight), and CD34⁺ cell dose (×10^6^/kg recipient weight).
3. Transplantation procedure and supportive care: Conditioning regimen type [myeloablative conditioning [MAC], reduced-intensity conditioning [RIC], MAC were defined as regimens containing busulfan ≥3.8 mg/kg or cyclophosphamide ≥60 mg/kg, and RIC were defined as regimens with lower doses or non-myeloablative agents], use of antithymocyte globulin (ATG) or alemtuzumab, GVHD prophylaxis regimen (cyclosporine A combined with methotrexate, mycophenolate mofetil), stem cell infusion date, time to neutrophil engraftment [days from transplantation to absolute neutrophil count (ANC) ≥ 0.5 × 10^9^/L for 3 consecutive days], and stem cell infusion indicators (CD34 + cell infusion quantity, T cell infusion quantity).

### Outcome variables

The primary outcome was the development of acute cutaneous GVHD within 100 days after allo-HSCT. Acute GVHD was diagnosed and graded according to the International Bone Marrow Transplant Registry (IBMTR) criteria (20), with cutaneous GVHD defined as the presence of maculopapular rash (without other identifiable causes such as drug eruption, infection, or underlying skin disease) involving the skin (21). The severity of cutaneous GVHD was classified as grade I [rash involving <25% body surface area (BSA)], grade II (rash involving 25%–50% BSA), grade III (rash involving 51%–75% BSA), or grade IV [rash (>50%) accompanied by blister formation or epidermal desquamation (>5%)] (22). Diagnosis was confirmed by clinical assessment, and skin biopsy was performed in cases with ambiguous clinical manifestations to exclude other etiologies.

### Statistical analysis

Statistical analysis was performed using SPSS 26.0 software (IBM Corp., Armonk, NY, USA). Quantitative variables were described as mean ± standard deviation (SD) or median [interquartile range (IQR)] based on normality (assessed by the Shapiro–Wilk test). Categorical variables were presented as *n* (%).​ Univariate analysis was used to identify potential risk factors for acute cutaneous GVHD. For quantitative variables, comparisons between the acute cutaneous GVHD group and non-GVHD group were performed using the independent samples *t*-test (normal distribution) or Mann–Whitney *U*-test (non-normal distribution). For categorical variables, the *χ*^2^-test or Fisher's exact test (when expected frequency was used.​ Logistic regression was adopted for binary endpoint analysis at day 100 rather than time-to-event competing risk models, and this analytical choice was justified by two cohort-specific characteristics: the highly concentrated early onset of acute cutaneous GVHD and the extremely low cumulative incidence of competing non-relapse mortality within 100 days post-transplant. Variables with a *P*-value < 0.1 in univariate analysis were included in the multivariate logistic regression model to adjust for confounding factors. The results of logistic regression were presented as odds ratios (ORs) and 95% confidence intervals (CIs). A two-tailed *P*-value was considered statistically significant. To assess the robustness of the results, sensitivity analysis was implemented after excluding three patients with missing data, which accounted for less than 5% of the total cohort. Missing information was limited to partial graft T-cell subset counts and immunosuppressive drug trough levels, and missingness was judged to be random and unrelated to the occurrence of acute cutaneous GVHD. Consistent multivariate regression outputs were obtained after case exclusion, which verified that missing records did not induce substantial bias to the primary analytical results. Additionally, subgroup analyses were performed based on skin GVHD grading.

## Results

### Baseline characteristics of the study population

**Figure 1:**
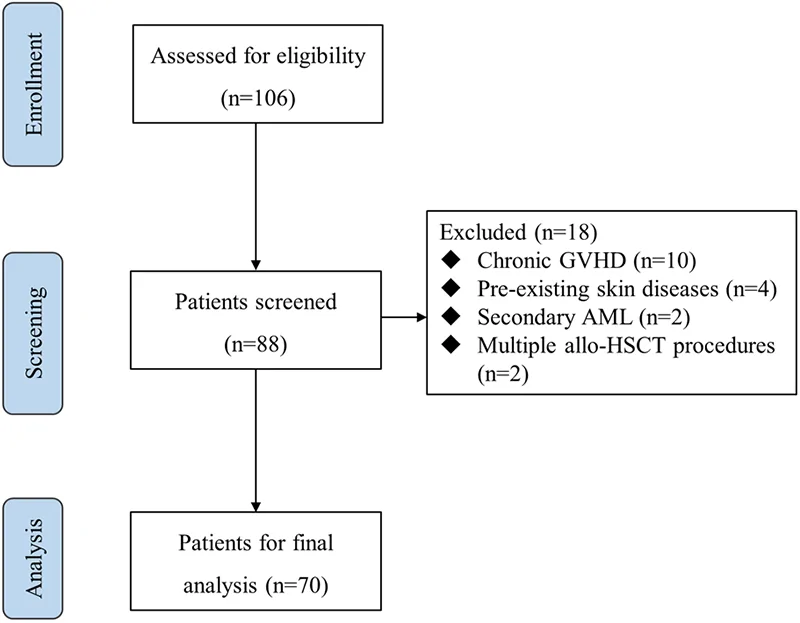
The study flowchart.

A total of 70 AML patients (age <18 years, median: 9.2 years) who underwent first allo-HSCT were finally enrolled. The flowchart for patient inclusion and exclusion is shown in Figure 1. There were 40 males (57.1%) and 30 females (42.9%), with a mean BMI of (18.5 ± 2.3) kg/m^2^. According to FAB classification, M2 was the most common subtype (31.4%). Most patients were in first complete remission (CR1, 78.6%) before transplantation, and 87.1% had a Charlson Comorbidity Index (CCI) score of 0.

Regarding transplant-related factors: HLA-identical sibling donors (ISD) accounted for 65.7%, HLA-matched unrelated donors (MUD) 17.1%, and haploidentical donors 17.1%; 61.4% of donor-recipient pairs were ≥8/10 HLA-matched. Peripheral blood stem cells (PBSC) were the main stem cell source (68.6%). Myeloablative conditioning (MAC) regimens were used in 70.0% of patients, and cyclosporine A combined with methotrexate was the primary GVHD prophylaxis (60.0%). The median time to neutrophil engraftment was 15 days (Table 1).

**Table 1:** Baseline characteristics of 70 AML patients undergoing allo-HSCT.

Characteristics	Value (*n* = 70)
Gender (male/female), *n* (%)	40 (57.1)/30 (42.9)
Age, median (range), years	9.2 (2.3–17.8)
BMI, mean ± SD, kg/m^2^	18.5 ± 2.3
AML subtype (FAB), *n* (%)	M1:18 (25.7); M2:22 (31.4); M3:10 (14.3); M4:8 (11.4); M5:7 (10.0); Others:5 (7.1)
Disease status at transplant, *n* (%)	CR1:55 (78.6); CR2:10 (14.3); Refractory/relapsed:5 (7.1)
Donor type, *n* (%)	ISD:46 (65.7); MUD:12 (17.1); Haploidentical:12 (17.1)
HLA mismatch (≥2/10/<8/10), *n* (%)	27 (38.6)/43 (61.4)
Stem cell source, *n* (%)	PBSC:48 (68.6); BM:17 (24.3); Cord blood:5 (7.1)
Conditioning regimen (MAC/RIC), *n* (%)	49 (70.0)/21 (30.0)
GVHD prophylaxis, *n* (%)	CsA + MTX:42 (60.0); CsA + MMF:28 (40.0)
Neutrophil engraftment time, median (range), days	15 (10–28)

AML, acute myeloid leukemia; allo-HSCT, allogeneic hematopoietic stem cell transplantation; BMI, body mass index; FAB, French-American-British; CR1, first complete remission; CR2, second complete remission; ISD, HLA-identical sibling donor; MUD, HLA-matched unrelated donor; HLA, human leukocyte antigen; PBSC, peripheral blood stem cells; BM, bone marrow; MAC, myeloablative conditioning; RIC, reduced-intensity conditioning; CsA, cyclosporine A; MTX, methotrexate; MMF, mycophenolate mofetil.

### Incidence and grading of acute cutaneous GVHD

Among 70 patients, 27 developed acute cutaneous GvHD within 100 days after transplantation, with an overall incidence of 38.6%. According to IBMTR criteria, the distribution of grades among the 27 patients with acute cutaneous GVHD was as follows: grade I (14.8%, 4/27), grade II (59.3%, 16/27), grade III (25.9%, 7/27), and no grade IV cases. Grade II was the most common subtype (Table 2).

**Table 2:** Grading distribution of acute cutaneous GVHD.

**aGvHD Grade**	**n**	**Proportion of aGvHD Patients (%)**	**Proportion of Total Patients (%)**
I	4	14.8	5.7
II	16	59.3	22.9
III	7	25.9	10.0
IV	0	0.0	0.0
Total	27	100.0	38.6

### Clinical outcomes related to acute cutaneous GVHD

The onset time of acute cutaneous *GVHD* in the 27 patients ranged from 14 to 42 days after transplantation, with a median onset time of 24 days. Stratified by grading, the median onset time of grade I acute cutaneous GvHD was 31 days (range: 25–38 days), grade II was 23 days (range: 16–32 days), and grade III was 18 days (range: 14–25 days). The onset time showed a trend of advancing with the increase of acute cutaneous GvHD grading, suggesting that severe acute cutaneous GvHD may occur earlier after transplantation.

All 27 acute cutaneous GvHD patients received first-line glucocorticoid therapy (methylprednisolone 2 mg/kg/d). After 2 weeks of treatment, the therapeutic response was evaluated: 18 cases (66.7%) achieved complete remission (CR), 7 cases (25.9%) achieved partial remission (PR), and 2 cases (7.4%) showed no response (NR). The therapeutic response rate (CR + PR) was 92.6%. Stratified by acute cutaneous GvHD grading, the complete remission rate of grade I patients was 100.0% (4/4), grade II was 75.0% (12/16), and grade III was 28.6% (2/7). The difference in therapeutic response among different grading groups was statistically significant (*χ*^2^ = 14.28, *P* < 0.001), indicating that the higher the acute cutaneous GvHD grading, the lower the sensitivity to glucocorticoid therapy (Table 3).

**Table 3:** Therapeutic response of aGvHD by grading.

**aGvHD Grade**	**CR (n, %)**	**PR (n, %)**	**NR (n, %)**	**Response Rate (%)**
I	4 (100.0)	0 (0.0)	0 (0.0)	100.0
II	12 (75.0)	3 (18.8)	1 (6.2)	93.8
III	2 (28.6)	4 (57.1)	1 (14.3)	85.7
Total	18 (66.7)	7 (25.9)	2 (7.4)	92.5
Statistical value	–	–	–	*χ*^2^ = 14.28, *P* < 0.001

### Risk factors for acute cutaneous GVHD

Univariate analysis was performed to explore the correlation between various baseline characteristics and the occurrence of acute cutaneous GVHD. The results showed that donor type (*χ*^2^ = 26.983, *P* < 0.001), donor-recipient HLA matching status (*χ*^2^ = 11.037, *P* = 0.001), disease status at transplantation (*χ*^2^ = 15.092, *P* = 0.001), and conditioning regimen type (*χ*^2^ = 4.825, *P* = 0.028) were significantly associated with the occurrence of acute cutaneous GVHD. In addition, gender, age, BMI, AML subtype, ABO blood group compatibility, stem cell source, infused TNC/CD34⁺ cell dose, use of ATG/alemtuzumab, and GVHD prophylaxis regimen were not significantly correlated with the occurrence of acute cutaneous GvHD (all *P* > 0.05) (Table 4).

**Table 4:** Univariate analysis of risk factors for acute cutaneous GVHD.

Variables	Acute cutaneous GvHD Cases (n, %)	Non-acute cutaneous GvHD Cases (n, %)	*χ*²	*P* Value
Gender			0.045	0.832
Male	15 (55.6)	25 (58.1)		
Female	12 (44.4)	18 (41.9)		
Age at transplantation (years)			0.009	0.925
≤10	16 (59.3)	25 (58.1)		
>10	11 (40.7)	18 (41.9)		
BMI (kg/m²)			0.018	0.894
≤18	13 (48.1)	20 (46.5)		
>18	14 (51.9)	23 (53.5)		
AML subtype			0.045	0.997
M1/M2	15 (55.6)	25 (58.1)		
M3	4 (14.8)	6 (14.0)		
M4/M5	6 (22.2)	9 (20.9)		
Others	2 (7.4)	3 (7.0)		
Disease status at transplantation			15.092	0.001
CR1	15 (55.6)	40 (93.0)		
CR2	7 (25.9)	3 (7.0)		
Refractory/relapsed	5 (18.5)	0 (0.0)		
Donor type			26.983	<0.001
ISD	8 (29.6)	38 (88.4)		
MUD	8 (29.6)	4 (9.3)		
Haploidentical donor	11 (40.7)	1 (2.3)		
Donor-recipient HLA mismatch			11.037	0.001
<8/10	10 (37.0)	33 (76.7)		
≥2/10 alleles	17 (63.0)	10 (23.3)		
Conditioning regimen type			4.825	0.028
MAC	23 (85.2)	26 (60.5)		
RIC	4 (14.8)	17 (39.5)		
ABO blood group compatibility			0.518	0.914
Compatible	11 (40.7)	21 (48.8)		
Minor incompatible	8 (29.6)	10 (23.3)		
Major incompatible	6 (22.2)	9 (20.9)		
Bidirectional incompatible	2 (7.4)	3 (7.0)		
Use of ATG/alemtuzumab			0.000	1.000
Yes	17 (63.0)	27 (62.8)		
No	10 (37.0)	16 (37.2)		
Stem cell source			0.077	0.962
PBSC	18 (66.7)	30 (69.8)		
BM	7 (25.9)	10 (23.3)		
Cord blood	2 (7.4)	3 (6.9)		
Infused TNC dose (×10⁸/kg)			0.544	0.461
≤8.6 (median)	12 (44.4)	23 (53.5)		
>8.6	15 (55.6)	20 (46.5)		
Infused CD34⁺ cell dose (×10^6^/kg)			1.508	0.219
≤5.2 (median)	11	24		
>5.2	16	19		
GVHD prophylaxis regimen			0.010	0.921
Cyclosporine A + methotrexate	16 (59.3)	26 (60.5)		
Cyclosporine A + mycophenolate mofetil	11 (40.7)	17 (39.5)		

GvHD, graft-versus-host disease; BMI, body mass index; AML, acute myeloid leukemia; TNC, total nucleated cell; PBSC, peripheral blood stem cells; BM, bone marrow; CR1, first complete remission; CR2, second complete remission; ISD, HLA-identical sibling donor; MUD, HLA-matched unrelated donor; HLA, human leukocyte antigen; MAC, myeloablative conditioning; RIC, reduced-intensity conditioning; ATG, antithymocyte globulin.

Variables with *P* < 0.1 in univariate analysis (donor type, donor-recipient HLA matching status, disease status at transplantation, conditioning regimen type) were included in the multivariate logistic regression model to adjust for confounding factors. The results showed that donor-recipient HLA mismatch ≥2/10 alleles (OR = 4.26, 95% CI: 1.68–10.82, *P* = 0.002), refractory/relapsed disease status at transplantation (OR = 6.83, 95% CI: 1.57–29.65, *P* = 0.011), and MAC regimens (OR = 3.15, 95% CI: 1.08–9.19, *P* = 0.036) were independent risk factors for the occurrence of acute cutaneous GVHD (Table 5). It should be noted that the wide confidence interval for the refractory/relapsed disease variable reflects limited precision in effect estimation, driven by the small number of events in this subgroup (*n* = 5, all in the aGVHD cohort), and caution against overinterpreting the magnitude of this point estimate.

**Table 5:** Multivariate logistic regression analysis of risk factors for acute cutaneous GVHD.

**Variables**	**Odds Ratio (OR)**	**95% Confidence Interval (CI)**	***P* Value**
Donor-recipient HLA mismatch (≥2/10 alleles vs. <8/10)	4.26	1.68–10.82	0.002
Disease status at transplantation (refractory/relapsed vs. CR1)	6.83	1.57–29.65	0.011
Conditioning regimen (MAC vs. RIC)	3.15	1.08–9.19	0.036
Donor type (haploidentical vs. ISD)	2.78	0.95–8.10	0.062

Reference group for disease status at transplantation is CR1; reference group for donor type is ISD. HLA, human leukocyte antigen; MAC, myeloablative conditioning; RIC = reduced-intensity conditioning; ISD, HLA-identical sibling donor; CR1, first complete remission.

### Other early post-transplant outcomes

Among the 70 patients, the cumulative incidence of non-relapse mortality (NRM) within the first 100 days post-transplant was 2.9% (*n* = 2). The causes of early mortality were sepsis (*n* = 1) and multiorgan failure secondary to veno-occlusive disease/sinusoidal obstruction syndrome (VOD/SOS) (*n* = 1). Primary graft failure, defined as the failure to achieve an absolute neutrophil count (ANC) > 0.5 × 10^9^/L by day +28 in the absence of relapse, occurred in 1 patient (1.4%). No cases of graft rejection were observed in this cohort.

### Sensitivity and subgroup analyses

Sensitivity analysis was performed by excluding 3 patients with missing data (≤5% of total variables), and the multivariate logistic regression model was re-run. The results showed that the independent risk factors identified remained consistent (donor-recipient HLA matching status <8/10: OR = 4.18, 95% CI: 1.62–10.89, *P* = 0.003; refractory/relapsed disease status: OR = 6.57, 95% CI: 1.48–29.13, *P* = 0.013; MAC regimens: OR = 3.09, 95% CI: 1.03–9.27, *P* = 0.044), indicating that the study results were robust. Subgroup analysis based on acute cutaneous GVHD grading showed that HLA mismatch at ≥2/10 alleles and refractory/relapsed disease status were more strongly associated with the occurrence of grade II-III acute cutaneous GVHD (HLA mismatch at ≥2/10 alleles: OR = 5.32, 95% CI: 1.87–15.12, *P* = 0.002; refractory/relapsed: OR = 8.15, 95% CI: 1.76–37.71, *P* = 0.007). In contrast, no statistically significant associations were observed between these risk factors and grade I acute cutaneous GVHD (all *P* > 0.05). However, this null finding for grade I aGVHD must be interpreted with caution given the extremely small number of grade I cases (*n* = 4), which rendered this subgroup analysis severely underpowered to detect clinically meaningful effect sizes, even if present.

## Discussion

This retrospective cohort study specifically investigated the clinical characteristics and risk factors for acute cutaneous GVHD in a homogeneous cohort of pediatric AML patients following their first allo-HSCT. Our findings demonstrate that donor-recipient HLA matching of less than 8/10, refractory or relapsed disease status at transplantation, and the use of a myeloablative conditioning regimen are independent risk factors significantly associated with the development of acute cutaneous GVHD. The overall low incidence of early non-relapse mortality (2.9%) and primary graft failure (1.4%) in our cohort suggests that the identified risk factors for acute cutaneous GVHD are not merely surrogates for overall poor engraftment or early survival but represent specific predictors for this immunologic complication. These results were robust upon sensitivity analysis and showed a particularly strong association with more severe (grade II-III) disease, providing valuable insights for risk stratification in this specific patient population.

HLA matching is the cornerstone of immune compatibility in allo-HSCT, and its mismatch directly drives alloreactive responses targeting the skin. When HLA mismatch at ≥2/10 alleles, mismatched alleles (HLA-A, -B, -C, -DRB1, -DQB1) trigger donor T cell activation via recipient antigen-presenting cells, leading to effector T cell infiltration into skin tissue and epithelial cell damage—manifesting as maculopapular rash or erythema (23). Pro-inflammatory cytokines (IFN-γ, complement component C3) released during this process amplify tissue injury (24), which aligns with our finding that this factor is strongly associated with grade II-III GVHD. Unlike some studies that only confirmed HLA mismatch as a general GVHD risk factor (25), our subgroup analysis specifies that the adverse effect is concentrated in moderate-to-severe cutaneous GVHD, likely due to cumulative allele mismatches inducing more sustained immune attacks. Notably, our results exclude minor HLA mismatches (≥8/10) from significant risk, suggesting a threshold effect consistent with IBMTR guidelines but more specific to pediatric AML cohorts.

Refractory/relapsed AML increases cutaneous GVHD risk through immune dysregulation and inflammatory priming (26). Long-term chemotherapy impairs regulatory T cell immunity function, losing control over donor T cell overactivation (27)—consistent with our finding that this status is the strongest independent risk factor. Additionally, persistent tumor cells may express cross-reactive antigens with skin tissues, triggering bystander immune responses, while pre-transplant systemic inflammation enhances donor T cell migration to the skin (28). Our study differs from prior mixed-malignancy research by showing a more pronounced effect in pediatric patients: their immature immune systems are less able to tolerate pre-existing inflammation, explaining why refractory/relapsed status correlates more strongly with severe cutaneous GVHD than in adult cohorts. This highlights the need for age-specific risk stratification.

MAC regimens elevate cutaneous GVHD risk via skin barrier disruption and inflammatory amplification (29). High-dose agents induce keratinocyte apoptosis and release damage-associated molecular patterns (HMGB1, ATP), activating local antigen-presenting cells and enhancing donor T cell recruitment (30)—consistent with our observation that MAC is an independent risk factor. Meanwhile, our results show MAC's effect is attenuated in patients receiving ATG, suggesting immunosuppressive prophylaxis can offset conditioning-related inflammation (31). Additionally, our pediatric-focused data reveals a low MAC-associated GVHD incidence, likely due to pediatric skin's stronger repair capacity and faster immune reconstruction, which mitigates long-term tissue damage (32).

The risk factors identified in our study resonate with, yet also distinguish themselves from, findings in prior studies on cutaneous GVHD. Consistent with the broader literature, HLA mismatch is a well-established driver of alloreactivity. For instance, Vargas-Díez et al. (11) also identified HLA disparity as a significant risk factor for acute cutaneous GVHD in a mixed hematological disorder cohort. Similarly, the association of myeloablative conditioning (which often includes total body irradiation) with increased GVHD risk aligns with findings in both acute and chronic GVHD settings (33). However, our study provides a more focused lens. Unlike the study by González-Cruz et al. (10) which reported unrelated donor as a risk factor for severe acute cutaneous GVHD in a general pediatric HSCT population, our multivariate analysis in a homogeneous pediatric AML cohort did not retain donor type as an independent predictor after adjusting for HLA matching and disease status. This suggests that in pediatric AML, the immunogenetic mismatch captured by HLA typing may be a more precise risk indicator than the broader donor category. Furthermore, while Vargas-Díez et al. (11) highlighted hematological disease type (chronic myeloid leukemia) as a risk factor, our study specifies that within pediatric AML, the statusof the disease (refractory/relapsed) rather than the AML subtype itself, is critical. This refines risk stratification from disease classification to disease biology and tumor microenvironment at transplant. Importantly, our findings contrast with some risk factors reported for chronic cutaneous GVHD in children, underscoring the potential distinct pathophysiology between acute and chronic forms. For example, studies on ccGVHD have identified older age, peripheral blood stem cell source, and male sex as significant risk factors (34), none of which were associated with acute cutaneous GVHD in our cohort. This divergence reinforces the necessity of studying acute and chronic cutaneous GVHD as separate entities with potentially different risk profiles, even within the pediatric population.

This study provides several important contributions to the literature on GVHD in children, with its core value lying in addressing critical gaps in disease- and age-specific evidence rather than identifying novel risk factors. By focusing exclusively on a homogeneous pediatric AML cohort, we clarify which established GVHD risk determinants retain independent predictive value in this distinct population—addressing the longstanding limitation of prior studies that pooled heterogeneous hematologic malignancies or combined pediatric and adult patients. While the independent risk factors identified (HLA mismatch, refractory/relapsed disease, myeloablative conditioning) are well-described in broader GVHD literature, our findings provide the first rigorous validation of their relative importance specifically in pediatric AML, where unique disease biology and immune ontogeny may modify risk profiles. In addition, this study confirms a gradient correlation between these risk factors and GVHD severity (particularly the stronger association with grade II-III disease), offering mechanistic insights into how pre-transplant disease status and conditioning intensity interact to drive severe cutaneous alloreactivity in this population. The identification of independent risk factors provides an initial, experience-based framework for clinical risk stratification and individualized prevention in pediatric AML transplant recipients, though we acknowledge these recommendations remain generic and have not yet been translated into a validated risk score or clinical decision algorithm. For patients with high-risk features, clinicians may consider targeted preventive strategies with attention to institutional feasibility and resource availability: first, optimizing GVHD prophylaxis (e.g., augmenting ATG dosing or incorporating novel immunomodulators) (35)—noting that such intensification must balance efficacy with risks of infection and delayed immune reconstitution, particularly in resource-limited settings; second, strengthening preemptive monitoring with regular skin examinations during the early high-risk window (14–24 days post-transplant), supplemented by immune function testing where accessible, to enable early intervention (36); third, individualizing conditioning intensity by considering a reduction to reduced-intensity conditioning for multi-high-risk patients when disease status permits, to mitigate tissue damage and inflammatory priming while maintaining anti-leukemic efficacy. We further recognize that cost-effectiveness and implementation barriers (e.g., access to advanced immune monitoring, availability of second-line agents for steroid-refractory disease) vary widely across clinical settings, and these factors must be weighed alongside risk profiles in shared decision-making. Finally, the observed association between GVHD grading and therapeutic response supports timely treatment escalation for grade III disease refractory to glucocorticoids (e.g., transitioning to JAK inhibitors or other targeted therapies) (37), though access to such agents remains a global equity consideration. Prospective studies integrating these risk factors into a simple, cost-conscious risk scoring tool are warranted to translate these findings into actionable, universally applicable clinical algorithms.

This study has several limitations. First, as a single-center retrospective cohort study, the findings were inevitably influenced by institutional standardized transplant protocols, unified GVHD prophylaxis schemes and localized clinical management routines, and cross-center generalizability was therefore restricted. Rigorous inclusion and exclusion criteria were strictly standardized, and sensitivity analyses were conducted to validate the robustness of multivariate regression outputs. Nevertheless, inherent selection bias and unmeasured confounders cannot be fully eliminated by retrospective data collection. Second, although we analyzed a comprehensive set of variables based on prior literature, the retrospective design inherently limited our ability to assess all potential modifiers of aGVHD risk. Most notably, we lacked systematic data on the precise trough levels and kinetic variability of calcineurin inhibitors and other immunosuppressive agents, as well as the incidence, magnitude, and kinetics of viral reactivations—particularly CMV and HHV-6, which are established triggers of alloreactive immune responses and central mediators of aGVHD pathogenesis. Other unmeasured confounders, including detailed donor-recipient KIR ligand mismatches and serial proinflammatory cytokine profiles, further limit our model's ability to account for all variance in aGVHD risk. Third, the application of logistic regression instead of competing risk time-to-event models represented a methodological limitation of the present analysis. The proportional hazard assumption was not assessed, and competing risks including early non-relapse mortality were not formally adjusted via Fine-Gray models. Only the binary presence or absence of acute cutaneous GVHD by day 100 was evaluated, while the impact of the identified risk factors on the timing of aGVHD onset could not be quantified using the current analytical framework. Larger cohorts with dense serial follow-up records are required in future investigations to enable competing risk regression and proportional hazard assumption testing, through which dynamic time-dependent risk associations can be comprehensively characterized. Fourth, while the 70-patient cohort met the *post-hoc* power threshold, merely 27 cutaneous aGVHD events were captured. Small subgroups (refractory/relapsed AML, *n* = 5; grade III aGVHD, *n* = 7) compromised multivariate model stability and yielded wide confidence intervals that reduced effect estimate precision. Detailed subgroup and interaction analyses were omitted due to insufficient statistical power. Larger multicenter cohorts are needed to validate these findings and support robust stratified analyses. Notably, the very small number of patients with refractory/relapsed disease (*n* = 5, all of whom developed aGVHD) raises concerns about potential overfitting and instability in the corresponding odds ratio estimate. While the association reached statistical significance, the extremely wide 95% CI underscores that this finding should be interpreted as hypothesis-generating rather than a precise quantification of risk, and requires validation in larger multicenter cohorts.

## Conclusion

In conclusion, this study confirms that HLA disparity, active disease at transplant, and MAC are significant predictors of acute cutaneous GVHD in pediatric AML patients undergoing allo-HSCT. These findings support the implementation of optimized risk stratification before transplantation. For pediatric patients with these risk factors, more aggressive or novel GVHD prophylaxis regimens, intensified monitoring for early signs of skin involvement, and potentially preemptive interventions warrant investigation in future prospective studies. Future prospective multicenter validation studies with complete recording of viral reactivation and lymphocyte subset biomarkers are required to corroborate our findings and improve the generalizability of pediatric AML-specific cutaneous aGVHD risk stratification strategies. Moreover, competing risk survival models with proportional hazard assumption testing should be adopted in subsequent prospective multicenter analyses to further elaborate time-dependent risk profiles of acute cutaneous GVHD.

## Data Availability

The original contributions presented in the study are included in the article/Supplementary Material, further inquiries can be directed to the corresponding author.
