# Correction: Clinical characteristics and risk factors of acute cutaneous graft-vs.-host disease following allogeneic hematopoietic stem cell transplantation in pediatric acute myeloid leukemia: a singlecenter retrospective study

**DOI:** 10.3389/fped.2026.1967080

**Published:** 2026-09-21

**Authors:** Shan He, Mincui Zheng, Wenyong Kuang, Na Song, Shaoyang Deng, Benshan Zhang

**Affiliations:** Department of Hematology/Oncology, The Affiliated Children’s Hospital of Xiangya School of Medicine, Central South University (Hunan Children’s Hospital), Changsha, China

**Keywords:** acute myeloid leukemia, graft-vs.-host disease, hematopoietic stem cell transplantation, pediatric, risk factors

Text correction

The originally reported recruitment period “July 1, 2022 and December 31, 2024” is incorrect. The actual consecutive patient recruitment period for this single-center retrospective study is December 2019 to September 2025.

A correction has been made to the section 1. Abstract, Methods subsection: revise the enrollment time window from “July 1, 2022 and December 31, 2024” to “December 2019 and September 2025”. 2. Materials and Methods, Study Population subsection: revise the enrollment time window from “July 1, 2022 and December 31, 2024” to “December 2019 and September 2025”

The corrected text appears below.

“1. Methods: A retrospective cohort study included pediatric AML patients who underwent their first allo-HSCT at Hunan Children's Hospital between December 2019 and September 2025. Demographic, disease-related, donor-recipient matching, transplant procedure, and supportive care data were collected. The primary outcome was acute cutaneous GVHD within 100 days post-transplant. Univariate analysis identified potential risk factors, and multivariate logistic regression confirmed independent predictors, with sensitivity analysis verifying robustness. 2. The study cohort consisted of consecutive pediatric patients diagnosed with AML who underwent their first allo-HSCT at our institution between December 2019 and September 2025. Inclusion Criteria: (1) age <18 years; (2) Confirmed diagnosis of AML in accordance with the Fifth Edition of the World Health Organization (WHO) Classification of Tumors of the Hematopoietic and Lymphoid Tissues (14); (3) Complete medical records available, including demographic information, AML cytogenetic and molecular genetic data, transplant-related details, post-transplant follow-up records, and outcome information; (4) Follow-up duration ≥100 days after allo-HSCT (consistent with the time window for defining acute GVHD); (5) No history of other malignant tumors or severe autoimmune diseases before transplantation. Exclusion Criteria: (1) Secondary AML, such as transformed from myelodysplastic syndrome or after chemotherapy/radiotherapy for other diseases; (2) Received haploidentical allo-HSCT with incomplete donor-recipient HLA matching data; (3) Pre-existing skin diseases that could interfere with the diagnosis of acute cutaneous GVHD; (4) Received immune-modulating therapy within 3 months before allo-HSCT; (5) Multiple allo-HSCT procedures.”

The original version of this article has been updated.

